# Case report: Complete remission of bone metastasis from renal cell carcinoma in histopathological examination after treatment with immune checkpoint inhibitors

**DOI:** 10.3389/fimmu.2022.980456

**Published:** 2022-09-27

**Authors:** Yohei Asano, Norio Yamamoto, Katsuhiro Hayashi, Akihiko Takeuchi, Shinji Miwa, Kentaro Igarashi, Takashi Higuchi, Yuta Taniguchi, Sei Morinaga, Takashi Horimoto, Masaharu Nakai, Yoshifumi Kadono, Takayuki Nojima, Hiroyuki Tsuchiya

**Affiliations:** ^1^ Department of Orthopaedic Surgery, Kanazawa University Graduate School of Medical Sciences, Kanazawa, Japan; ^2^ Department of Orthopaedic Surgery, Kaga Medical Center, Kaga, Japan; ^3^ Department of Urology, Kaga Medical Center, Kaga, Japan; ^4^ Department of Integrative Cancer Therapy and Urology, Kanazawa University Graduate School of Medical Sciences, Kanazawa, Japan

**Keywords:** renal cell carcinoma (RCC), immune checkpoint inhibitor (ICI), nivolumab, bone metastasis (BM), pathological fracture

## Abstract

Recently, the prognosis of metastatic renal cell carcinoma (mRCC) has improved owing to the development of immunotherapy using immune checkpoint inhibitors (ICIs). However, there have been few studies on the therapeutic effect of ICIs in bone metastases from renal cell carcinoma (RCC). We report a case in which pulmonary and humeral metastases from RCC were significantly ameliorated using ICIs, while surgery for a pathological fracture of the humerus significantly improved the patient’s quality of life (QoL). A 70-year-old man who underwent a left nephrectomy for RCC developed multiple pulmonary metastases and humeral metastasis with a pathological fracture one year after surgery, and combined treatment with nivolumab and ipilimumab was initiated. After four courses of ICI treatment, multiple pulmonary metastases had almost disappeared, and the tumor at the fracture site had shrunk remarkably. However, the shoulder joint function had decreased due to the fracture, worsening his QoL. Therefore, he underwent surgery and returned to normal daily life one month after. Postoperative histopathological examination of bone and soft tissue at the fracture site revealed no malignancy. To our knowledge, this is the first case report of complete remission of bone metastasis of RCC based on histopathological examination with ICI treatment.

## Introduction

Among patients with renal cell carcinoma (RCC), 25–30% already have distant metastasis at the time of diagnosis (1, 2), and 30% of patients with metastatic RCC (mRCC) have bone metastasis (3). The 5-year overall survival (OS) rate for mRCC, including patients with bone metastasis, has been reported to be 12% (1, 2), and the prognosis is very poor. Bone metastatic lesions from RCC are osteolytic changes, and skeletal-related events (SRE), such as severe bone pain, pathological fractures, and spinal compression, occur in > 70% of patients with mRCC (4). The quality of life (QoL) and prognosis of patients with mRCC are worsened by SRE (4, 5), and bone metastasis is a poor prognostic factor (6–8). However, in recent years, the development of immune checkpoint inhibitors (ICIs) has improved the prognosis of mRCC in some clinical trials (9, 10), and ICIs have become the standard treatment (11). In mRCC patients with intermediate-risk/poor-risk by the International Metastatic RCC Database Consortium (IMDC) risk classification (12), the 5-year OS rate was dramatically improved to 43% with ICI treatment (13). Similarly, although improvement in median survival time of mRCC with bone metastases has been reported (14), there are few reports regarding the therapeutic effect of ICIs on bone metastases in RCC. We report the first case of multiple pulmonary and humeral metastases in a patient with RCC that was dramatically ameliorated by ICI treatment, and complete remission was observed on histopathological examination.

## Case presentation

**Figure 1:**
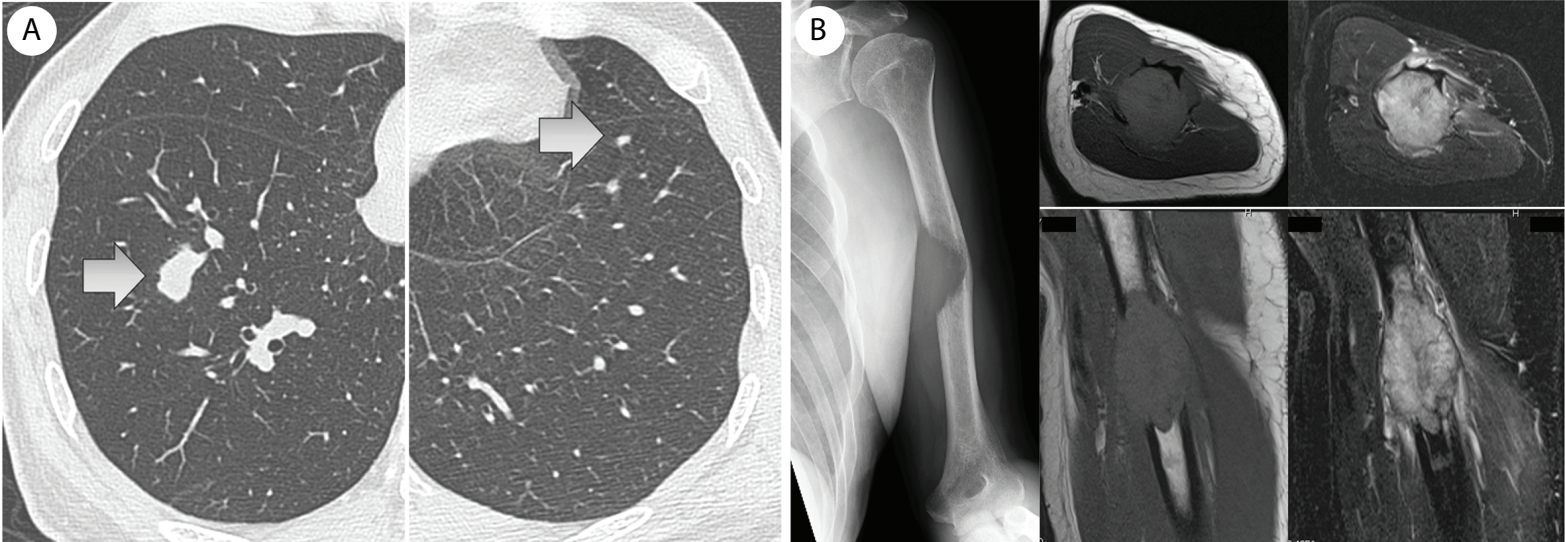
**(A)** One year after the left nephrectomy, two lung metastases were revealed by computed tomography. **(B)** Eight months later, a left humerus fracture with osteolytic change was revealed by X-ray. Magnetic resonance imaging (MRI) showed an extraskeletal mass at the fracture site (left: T1-weighted image, right: fat-suppressed T2-weighted image), and it was considered to be a pathological fracture due to bone metastasis.

**Figure 2:**
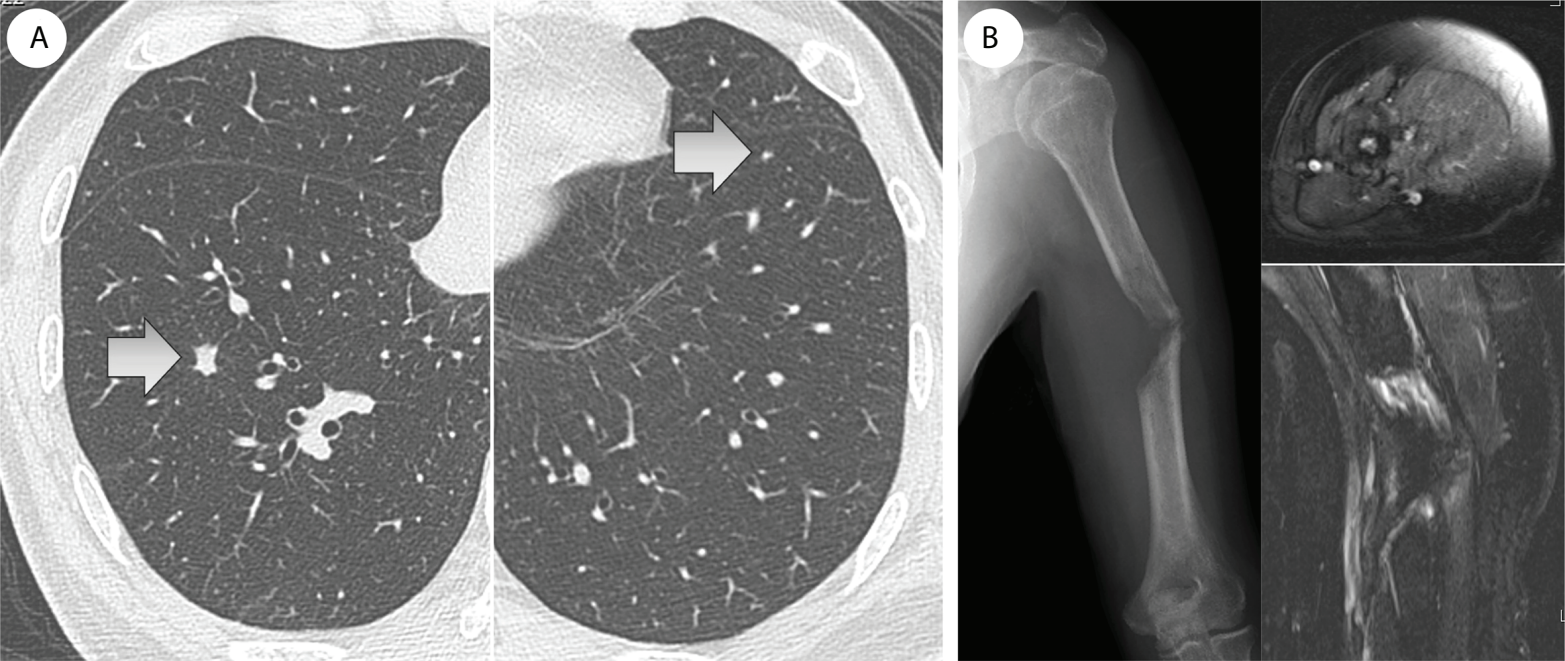
**(A)** After four courses of ICI treatment, the pulmonary metastases had almost disappeared. **(B)** Furthermore, the tumor at the fracture site had shrunk remarkably and almost disappeared (MRI: gadolinium-enhancement).

**Figure 3:**
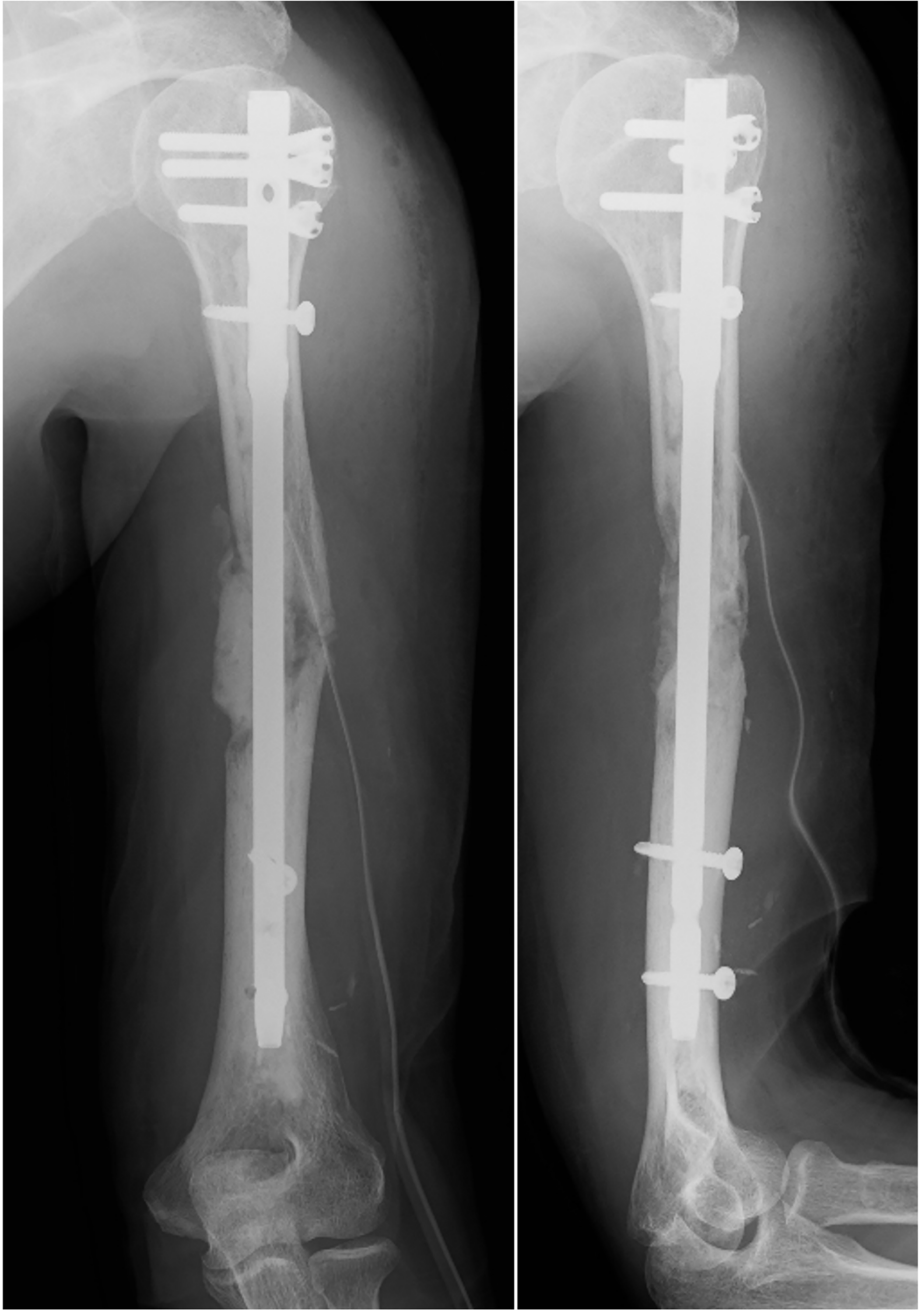
Postoperative X-ray.

**Figure 4:**
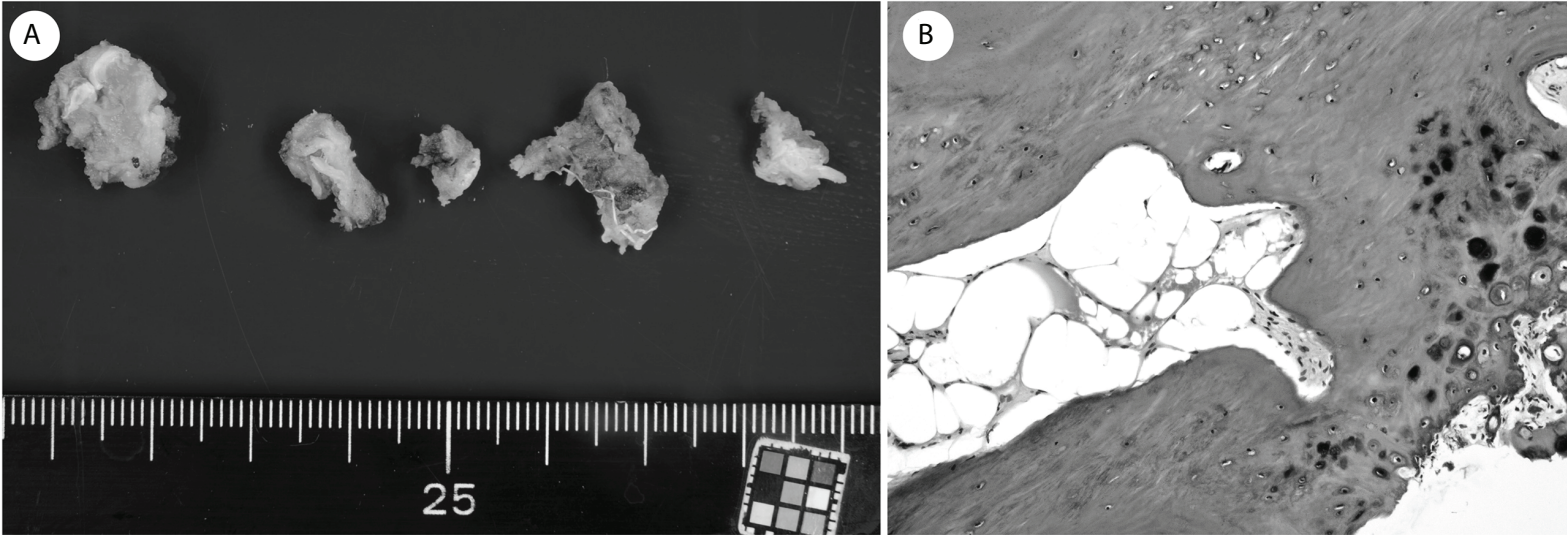
**(A)** The soft tissue at the fracture site. **(B)** Histopathological examination showed no evidence of malignancy, and the bone healing process after the fracture was observed.

A 70-year-old man diagnosed with RCC underwent a left nephrectomy because preoperative radiological examinations showed no distant metastasis and was followed up without drug treatment after the surgery. One year after the surgery, radiological examination revealed two lung metastases (**Figure 1A**), for which drug treatment was recommended, but the patient refused this. Eight months later, left upper arm pain appeared during his sleep, and X-ray radiographs revealed a fracture in the diaphysis of the humerus with osteolytic changes. Magnetic resonance imaging (MRI) showed a mass lesion extending to the outside of the fracture site, which was considered a pathological fracture due to bone metastasis (**Figure 1B**). He was clinically diagnosed with multiple metastases of RCC, and the IMDC risk classification (12) was evaluated as poor based on Karnofsky performance status < 80%, anemia, and high serum-corrected calcium. Since the pulmonary metastases had not been treated, ICI treatment using nivolumab and ipilimumab was immediately initiated to prioritize systemic treatment. The pathological fracture was treated conservatively with denosumab and concomitant functional brace fixation. After four courses of ICI treatment, the pulmonary metastases had almost disappeared (**Figure 2A**), and the therapeutic effect was evaluated as a partial response (PR) based on the Response Evaluation Criteria in Solid Tumors (RECIST) version 1.1 (15). Furthermore, the tumor at the pathological fracture site showed remarkable shrinkage (**Figure 2B**). However, the left shoulder joint function was significantly decreased due to the pathological fracture, and the active range of motion could not be evaluated. Since there was no metastasis to other sites, the pulmonary metastases had almost disappeared, and a long-term prognosis was expected, surgical treatment for pathological fracture was planned to improve shoulder joint function. Seven months after the diagnosis of pathological fracture, surgery was performed under general anesthesia. The soft tissue suspected that the tumor at the fracture site was resected, and curettage in the medullary cavity was performed to refresh the fracture site. The medullary cavity was then filled with cement, and an intramedullary nail was inserted. Finally, cement was added to the defect at the fracture site, and screw fixation of the nail was performed (**Figure 3**). Postoperatively, histopathological examination of the soft tissue at the fracture site and medullary cavity showed no evidence of malignancy, and bone healing after the fracture was observed (**Figure 4**). Based on the histopathological findings, humerus metastasis of RCC was evaluated as complete remission after ICI treatment. One month after the surgery, the pain of motion disappeared, and auto-flexion and abduction improved to 160° and 170°, respectively. To assess his postoperative QoL, the patient-reported outcomes using the disability of the arm, shoulder and hand (DASH) score (16) was performed and showed significant improvement (from 66.7 preoperatively to 20.0 postoperatively). The shoulder joint function and his QoL were greatly improved by the surgery for the pathological fracture. Seventeen months after the surgery, nivolumab monotherapy was continued without immune-related adverse events, and no recurrence or metastasis was observed. The patient has returned to his normal daily life and continues to be followed up with radiological examinations every six months.

## Discussion

Multiple pulmonary and humeral metastases from RCC were dramatically ameliorated by ICI treatment, and surgical treatment for the pathological fracture of the humerus remarkably improved shoulder joint function and QoL. To our knowledge, this is the first case report in which a bone metastatic lesion of RCC treated with ICIs was evaluated as complete remission based on histopathological examination.

Strategies for the treatment of mRCC have generally been considered based on the IMDC risk classification (12). Currently, in pharmacological therapy of mRCC, the ICI treatment using nivolumab and ipilimumab is approved as the first-line treatment for the patient evaluated as intermediate or poor in IMDC risk classification. The combined treatment with these ICIs significantly improved the 12-month overall survival rate (80% vs. 72%, *p*<0.001) and response rate (42% vs. 27%, *p*<0.001) for mRCC compared to the conventional standard treatment group treated with sunitinib in a phase III study (CheckMate 214) (9). The development of ICI treatment has brought a paradigm shift in the treatment strategy for patients with untreated advanced clear cell RCC who had insufficient therapeutic effects from conventional drug treatment and had poor prognoses (6, 7, 11).

Two case reports have shown that bone metastases from RCC were significantly improved by ICI treatment (17, 18). In both cases, nivolumab, which was introduced as a second-line treatment after the molecular-targeted drug, improved bone metastases. Vuyyala et al. (17) reported that a solitary scapular metastasis with osteolytic change was remodeled with osteosclerotic change by nivolumab treatment without the concomitant use of a bone-modifying agent (BMA). Marsh et al. (18) reported that the accumulation of a solitary thoracic vertebral body on positron emission tomography (PET) disappeared after nivolumab treatment. In both cases, although the therapeutic effect of nivolumab on bone metastasis was CR on radiological evaluation, no histopathological evaluation was performed. In our case, as in previous reports, remarkable amelioration of bone metastasis by ICI treatment was revealed on radiological examination. Furthermore, complete remission at the bone metastatic lesion was observed on histopathological examination, which is the first case report to evaluate the therapeutic effect of ICI on bone metastasis.

Bone metastasis in the long bones of the extremities is associated with a risk of pathological fracture, which is an SRE (19). In most cases, although non-surgical treatment including drug therapy with BMA and radiation therapy are considered (20, 21), surgical treatment may be performed for impending fractures based on the Mirels’ score (22) and for pathological fractures to improve function. In our case, ICI treatment significantly ameliorated multiple pulmonary and humeral metastases, and a long-term prognosis could be expected. However, a pathological fracture of the humerus significantly reduced the shoulder joint function and interfered with his daily life. Therefore, surgical treatment was performed to improve joint function and QoL. Postoperative histopathological examination revealed complete remission of bone metastasis with ICI treatment; thus, it was suggested that multidisciplinary therapy including ICIs may improve metastasis to the long bones in the extremities without surgery if pathological fracture can be prevented. For optimal management of long bone metastases to prevent SREs, including pathological fractures, early diagnosis of those metastases by radiological examinations is very important. After the diagnosis, careful conservative follow-up should be performed, such as fixation with functional bracing in the upper extremity and non-weight-bearing in the lower extremity. In addition, initiation of bone modifying agents such as denosumab may be recommended.

In our case, denosumab was used concomitantly, which might have affected its therapeutic effect on bone metastases. The effectiveness of combined treatment with ICIs and denosumab in bone metastases has been reported in melanoma and non-small cell lung cancer (23–27). Although there have been no reports on the combined treatment for bone metastases from RCC, denosumab has been licensed in treatment for mRCC and may be recommended the combined use.

## Conclusion

Multiple pulmonary and humeral metastases from RCC were dramatically ameliorated by ICI treatment with nivolumab and ipilimumab. Furthermore, surgical treatment for pathological fractures of the humerus remarkably improved shoulder joint function and QoL. To our knowledge, this is the first case report of complete remission of bone metastasis from RCC based on histopathological examination with ICI treatment. Multidisciplinary therapy with ICIs may influence the therapeutic strategies for mRCC with bone metastases.

## Data availability statement

The original contributions presented in the study are included in the article/supplementary material. Further inquiries can be directed to the corresponding author.

## Ethics statement

Written informed consent was obtained from the individual(s) for the publication of any potentially identifiable images or data included in this article.

## Author contributions

The manuscript was drafted by YA and NY. YA, NY, KH, AT, SMi, KI, TH, YT, SMo, TH, MN, YK and HT examined and treated the patient. TN performed the histopathological assessment. YA and NY analyzed data, and YA wrote the manuscript. NY and HT supervised this study. All authors contributed to the article and approved the submitted version.

## Acknowledgments

We would like to thank Editage (www.editage.com) for English language editing.

## Conflict of interest

The authors declare that the research was conducted in the absence of any commercial or financial relationships that could be construed as a potential conflict of interest.

## Publisher’s note

All claims expressed in this article are solely those of the authors and do not necessarily represent those of their affiliated organizations, or those of the publisher, the editors and the reviewers. Any product that may be evaluated in this article, or claim that may be made by its manufacturer, is not guaranteed or endorsed by the publisher.
